# Combined unilateral biportal endoscopy and video-assisted thoracoscopic surgery for complete excision of a T3–T4 right ganglioneuroma

**DOI:** 10.3171/2024.2.FOCVID23210

**Published:** 2024-04-01

**Authors:** Enrico Giordan, Changik Lee, Dimas Rahman Setiawan, Phattareeya Pholprajug, Jin-Sung Kim

**Affiliations:** 1 Spine Center, Department of Neurosurgery, Seoul St. Mary’s Hospital, College of Medicine, The Catholic University of Korea, Seoul, Korea;; 2 Department of Neurosurgery, AULSS2 Marca Trevigiana, Treviso, Italy;; 3 Faculty of Medicine, Universitas Indonesia, Jakarta, Indonesia; and; 4 Department of Orthopedics, Rayong Hospital, Rayong, Thailand

**Keywords:** ganglioneuroma, endoscopic surgery, minimally invasive surgery, biportal endoscopy, thoracoscopy

## Abstract

Ganglioneuroma (GN) is a rare solid neoplasm developing from neural crest cells of sympathetic ganglia or adrenal medulla. It usually presents as an asymptomatic mass in the retroperitoneal space and mediastinum. Resection through open surgery or minimal access is recommended. The video illustrates the case of a 23-year-old female with an incidental finding of thoracic GN. The authors performed a combined, staged approach to ensure complete resection, which involved unilateral T3–4 biportal endoscopy (UBE) for rhizotomy and nerve root decompression, followed by video-assisted thoracoscopic surgery (VATS) for complete excision. The procedure was uneventful, with full recovery and no postoperative complications.

The video can be found here: https://stream.cadmore.media/r10.3171/2024.2.FOCVID23210

## Transcript

The authors present a case of a combined unilateral biportal endoscopy and video-assisted thoracoscopic surgery for complete excision of a T3–4 right ganglioneuroma.

### 0:36 Background.

Ganglioneuroma is a rare tumor arising from neural crest cells of the sympathetic ganglia or adrenal medulla.^1^ It is a solid tumor at the benign end of the spectrum, occurring predominantly in children and adolescents.^2^ It often occurs in the retroperitoneal space and posterior mediastinum. Thoracic ganglioneuroma is sporadic. Because of the slow growth and benign nature, it is generally an asymptomatic mass found on routine chest radiography.^3^ The definitive treatment of ganglioneuroma is surgical resection, either by open surgery or by minimal access surgery when feasible.

### 1:15 Patient Presentation.

The patient is a 23-year-old female coming to our attention for an incidental diagnosis of right mediastinal mass. She has a negative past medical history and no complaints of sensory or neurological disturbances.

### 1:33 Imaging.

Imaging included chest x-rays and a CT of the thoracic spine, which showed a 4.4 for a 2-cm mass in the right paravertebral region with extension along the right posterior chest wall. Contrast-enhanced MRI showed the mass possibly arising from the T3–4 neuroforamen and T3 nerve root with a focal contrast enhancement suggestive of a neurogenic neoplasm.

### 2:04 Choosing the Surgical Strategy.

Surgery was deemed necessary for this patient and several strategies were evaluated. To achieve a complete resection, the first step was to detach the mass from the T3 nerve root by performing a rhizotomy. Various endoscopic approaches were considered, and ultimately biportal endoscopy was chosen over full-endoscopy due to the superior ability to use clips and scissors and operate with more degree of freedom. Then the remaining mass was completely excised through video-assisted thoracoscopic surgery.

### 2:38 Unilateral Biportal Endoscopy.

Unilateral biportal endoscopy procedure explanation.

### 2:45 Patient Positioning.

Under general anesthesia and neurophysiological monitoring, the patient was positioned prone on a Wilson frame. As always, marking levels preoperatively is done carefully with specific alternate anteroposterior and lateral-lateral fluoroscopic images.

### 3:04 Planning and Marking.

Key anatomical lines are drawn on the patient to mark the midline, the disc level, and the T3 and T4 pedicle as for a typical unilateral biportal approach. Contouring pedicles will highlight the limits of the upper and lower as well as the lateral and medial limits of the neuroforamen. Also, those are usual endoscopic and working port entry points.

### 3:29

Therefore, two incisions measuring 1.5 cm and spaced 2 cm apart are made for the endoscopic and working portals. That’s enough space to permit adequate triangulation of instruments to reach the foraminal area.

### 3:44

The muscle fascia is opened to allow for easier endoscope and working instrument positioning.

### 3:51

Serial dilation is performed in both incisions.

### 3:57

The endoscope and working instruments should converge to the target point with an angle of approximately 30°.

### 4:06

For right-handed surgeons, the left access is for endoscope maneuvering, while the right port is for instrument insertion and maneuvering.

### 4:15

Adequate outflow in biportal endoscopic surgery is confirmed to prevent iatrogenic neural injury, reduce bleeding, and enhance visualization.

### 4:26 Tissue Exposure.

The endoscope was introduced, and the surrounding tissue was removed and cauterized with a bipolar coagulator to reveal the underlying bony anatomy.

### 4:47 Drilling.

A 4-mm round diamond burr is used to remove bone. Progressive drilling of the superior articular process, lamina, and inferior articular process was performed to enlarge the space while keeping the ligamentum flavum intact.

### 5:06

After decreasing bone thickness, a Kerrison punch is used to cut the bone.

### 5:44 Root Finding.

Then, the ligamentum flavum is gently removed and the T3 nerve root is carefully revealed, with the tumor attached to its dorsal portion.

### 5:58

Posterior radicular artery clipping and sectioning for bleeding control.

### 6:11

Gentle separation of tumor from nerve root.

### 6:20 Intraoperative Nerve Stimulation.

Intraoperative nerve stimulation is used to confirm the T3 nerve root.

### 6:32 Rhizotomy.

The tumor was found in the nerve root’s dorsal part, and the root was subsequently gently coagulated and resected to allow for complete mass excision.

### 7:22 VAST.

Successively, the patient underwent video-assisted thoracoscopic surgery. The surgical position was changed to the left lateral position.

### 7:33

Although lung parenchyma was attached around the tumor, there were no clear signs of invasion. So, the two structures were separated relatively well using electrocautery.

### 7:48

The boundary of the tumor was examined, and the extent of resection was determined. The dissection was performed using a hook Bovie. The tumor was carefully dissected while preserving the intercostal nerve bundle.

### 8:02

Although an intercostal vessel injury occurred, bleeding control was achieved through compression and electrocautery.

### 8:51

The tumor was completely detached and removed using an Endo Bag.

### 9:09

After bleeding control, the thoracic surgery was completed without intraoperative adverse events.

### 9:20 Diagnosis.

The surgery led to the excision of a 3.3 × 2.2 × 2.2 mass. It was confirmed at the histopathological examination that the mass was a ganglioneuroma.

### 9:37 Postoperative Imaging.

The satisfactory and complete resection of the tumor was confirmed at postoperative spine CT and contrast-enhanced MRI, in which there were no signs of remnant neoplasm.

### 9:53 Postoperative Outcomes.

The procedure was uneventful, without perioperative adverse events occurrences. The patient returned to normal daily activities, with no late adverse events or recurrences at the 2-year follow-up. Uniportal bilateral endoscopy for rhizotomy and ganglioneuroma detachment provides comparable surgical outcomes to microsurgery and superior cosmetic results.

## Acknowledgments

We are grateful to Professor Seok Whan Moon for his expertise and collaboration in creating the video. His willingness to share his part of the operation is a valuable demonstration of the teamwork between neurosurgeons and thoracic surgeons. This partnership aims to enhance minimally invasive surgical techniques, ultimately improving patient care.

## Disclosures

The authors report no conflict of interest concerning the materials or methods used in this study or the findings specified in this publication.

## Author Contributions

Primary surgeon: Kim. Assistant surgeon: Lee. Editing and drafting the video and abstract: Giordan, Lee. Critically revising the work: Giordan, Lee. Reviewed submitted version of the work: all authors. Approved the final version of the work on behalf of all authors: Kim. Supervision: Kim, Giordan, Lee.

## Supplemental Information

### Patient Informed Consent

The necessary patient informed consent was obtained in this study.
